# A novel anti-galectin-9 immunotherapy limits the early progression of pancreatic neoplastic lesions in transgenic mice

**DOI:** 10.3389/fimmu.2023.1267279

**Published:** 2023-11-30

**Authors:** Alexandre Quilbe, Rami Mustapha, Belinda Duchêne, Abhishek Kumar, Elisabeth Werkmeister, Emmanuelle Leteurtre, Olivier Moralès, Nicolas Jonckheere, Isabelle Van Seuningen, Nadira Delhem

**Affiliations:** ^1^ Univ. Lille, Inserm, CHU Lille, U1189 - ONCO-THAI - Assisted Laser Therapy and Immunotherapy for Oncology, Lille, France; ^2^ Department of Cancer Studies and Pharmaceutical Sciences New Hunt’s House, School of Life Sciences and Medicine, King’s College London, London, United Kingdom; ^3^ Univ. Lille, CNRS, Inserm, CHU Lille, UMR9020-U1277 - CANTHER – Cancer Heterogeneity Plasticity and Resistance to Therapies, Lille, France; ^4^ Univ. Lille, CNRS, Inserm, CHU Lille, Institut Pasteur de Lille, US 41 - UMS 2014 -PLBS, Lille, France

**Keywords:** pancreatic adenocarcinoma, galectin-9, regulatory T cells, immunotherapy, transgenic mouse model, pancreatic intraepithelial neoplasm (PanIN)

## Abstract

**Background:**

Pancreatic adenocarcinoma (PDAC) is a devastating disease with an urgent need for therapeutic innovation. Immune checkpoint inhibition has shown promise in a variety of solid tumors, but most clinical trials have failed to demonstrate clinical efficacy in PDAC. This low efficacy is partly explained by a highly immunosuppressive microenvironment, which dampens anti-tumor immunity through the recruitment or induction of immunosuppressive cells, particularly regulatory T cells (Tregs). In this context, our laboratory has developed a novel immunotherapeutic strategy aimed at inhibiting the suppressive activity of Tregs, based on a patented (EP3152234B1) monoclonal antibody (mAb) targeting galectin-9 (LGALS9).

**Materials and methods:**

CD4+ conventional T cells (TCD4 or Tconv), Treg ratio, and LGALS9 expression were analyzed by immunohistochemistry (IHC) and cytometry in blood and pancreas of K-rasLSL.G12D/+;Pdx-1-Cre (KC) and K-rasWildType (WT);Pdx1-Cre (WT) mice aged 4–13 months. Pancreatic intraepithelial neoplasm (PanIN) progression and grade were quantified using FIJI software and validated by pathologists. The anti-galectin-9 mAb was validated for its use in mice on isolated murine C57BL/6 Treg by immunofluorescence staining and cytometry. Its specificity and functionality were validated in proliferation assays on rLGALS9-immunosuppressed murine Tconv and in suppression assays between murine Treg and Tconv. Finally, 2-month-old KC mice were treated with anti-LGALS9 and compared to WT mice for peripheral and infiltrating TCD4, Treg, and PanIN progression.

**Results:**

IHC and cytometry revealed a significant increase in LGALS9 expression and Treg levels in the blood and pancreas of KC mice proportional to the stages of precancerous lesions. Although present in WT mice, LGALS9 is expressed at a basal level with low and restricted expression that increases slightly over time, while Treg cells are few in number in their circulation and even absent from the pancreas over time. Using our anti-LGALS9 mAb in mice, it is shown that (i) murine Treg express LGALS9, (ii) the mAb could target and inhibit recombinant murine LGALS9, and (iii) neutralize murine Treg suppressive activity. Finally, the anti-LGALS9 mAb in KC mice reduced (i) LGALS9 expression in pancreatic cancer cells, (ii) the Treg ratio, and (iii) the total surface area and grade of PanIN.

**Conclusion:**

We demonstrate for the first time that an anti-LGALS9 antibody, by specifically targeting endogenous LGALS9 tumor and exogenous LGALS9 produced by Treg, was able to limit the progression of pancreatic neoplastic lesions in mice, opening up new prospects for its use as an immunotherapeutic tool in PDAC.

## Graphical Abstract

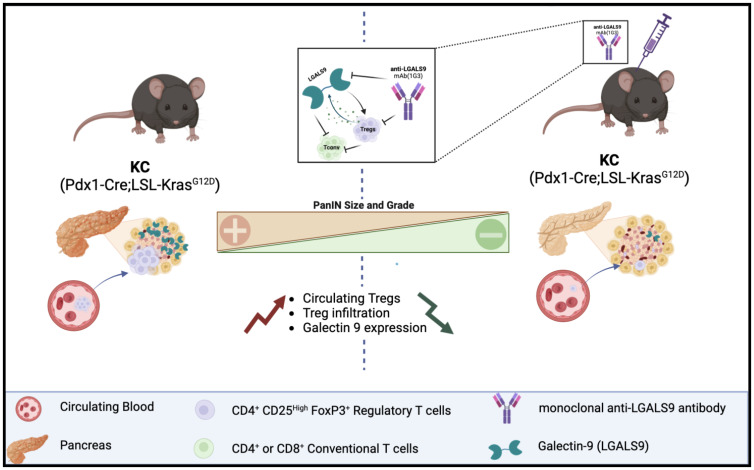
Summary of the conclusions of the study.

## Introduction

Pancreatic adenocarcinoma (PDAC) is a devastating disease for which the incidence is close to mortality (1, 2). It is estimated that it will be the second leading cause of cancer-related death in the United States and in Europe by 2030 (3, 4). PDAC is often diagnosed at an advanced stage because it progresses rapidly with few specific symptoms. Statistically, approximately 60% of the patients are diagnosed at the metastatic stage, nearly 30% at a locally advanced stage without metastasis and 15% at a surgically resectable stage (2, 5, 6). Adjuvant chemotherapy based on modified FOLFIRINOX protocol (poly-chemotherapeutic regimen composed of folinic acid, 5-FU, leucovorin, irinotecan, and oxaliplatin) is indicated for all patients after resection and offers 54.4 months overall survival (OS) (7). Currently, there is no established standard of care for unresectable/locally advanced and metastatic PDAC, where OS has been reported to be 15 months (with capecitabine-based chemotherapy) and 11 months (with FOLFIRINOX), respectively. No substantial improvements in treatment have been observed since FOLFIRINOX; only gemcitabine plus nab-paclitaxel were approved by the US Food and Drug Administration in 2011, and 2013, respectively. Finally, the 5-year overall survival (OS) rate does not exceed 11% (6, 8), and there is an urgent need for therapeutic innovation in order to improve the prognosis of this cancer.

Immune checkpoint inhibition (ICI) has shown promising results across multiple solid tumors and is now considered as a new standard in cancer treatment for many indications (9). Anti-programmed cell death protein 1 (PD1)/PD-ligand 1 (PD- L1) (pembrolizumab, atezolizumab) and anti-cytotoxic T-lymphocyte-associated protein 4 (CTLA-4) (ipilimumab) monotherapies were evaluated in PDAC patients; however, most phase I and II clinical trials have failed to show any clinical efficacy in PDAC (10–12). Nevertheless, in some subgroups of patients, ICI monotherapy has led to some good results. Indeed, an accumulation of studies indicates that PDAC patients with advanced microsatellite instability-high (MSI-H) or mismatch repair deficiency (dMMR) would benefit from anti-PD1 treatment (13–15). For instance, the KEYNOTE-158, a phase 2 study only including non-colorectal cancer patients with advanced MSI-H/dMMR of which 22 were PDAC patients, the overall response rate (ORR) was 18.2% with pembrolizumab, including one complete response (CR), three partial response (PR), and a median duration of response (mDOR) of 13.4 months (14). Moreover, in a series of retrospective data analysis, Amin and collaborators have examined the impact of immunotherapy on the overall survival (OS) of patients diagnosed with PDAC (2004–2016) using the US National Cancer Database (NCDB). They could assess a positive role of ICI for patients who received definitive surgery or not of tumor, since immunotherapy improved OS compared to patients who did not receive it (16, 17). The role of immunotherapy, whether neoadjuvant or adjuvant, remains to be defined (18, 19). Interestingly, combinations of therapeutic protocols using immune checkpoint inhibitors with radiotherapy and/or chemotherapy have shown encouraging results, suggesting that an effective immune response modulation is possible in PDAC (12, 20, 21). The low efficacy of checkpoint inhibitors in PDAC may be partly explained by its specificities, including its highly immunosuppressive microenvironment (22). Indeed, PDAC is usually described as a “cold” tumor regarding the poor effector T-cell infiltrate. Multiple components of PDAC tumor microenvironment (TME) dampen anti-tumor immunity through recruitment or induction of immunosuppressive cells particularly myeloid cell [tumor-associated macrophages (TAMs), granulocytes and inflammatory monocytes, and myeloid-derived suppressor cells (MDSCs)], suppressive B cells, immunosuppressive gamma delta T cells (γδ T cells), and regulatory T cells (Tregs), which restrain anti-tumor immune response (23–25).

In many cancers, including PDAC, Tregs are prominent components of the infiltrating and circulating T lymphocyte population strongly associated with poor prognosis and inversely correlated with the presence of effector T cells (26, 27). It is well described that Treg neutralization favours an immunostimulatory environment and is a major way to restore anti-tumor immunity (28, 29). In this context, our laboratory has developed a new immunotherapeutic strategy aimed at specifically inhibiting the suppressive activity of natural occurring regulatory T cells (CD4+ CD25high FoxP3+ CD127−/low). This strategy is based on a monoclonal antibody (mAb), namely, 1G3 (patent WO2015185875) targeting galectin-9 (LGALS9) lectin notably produced and used by Tregs to mediate immunosupression.

Genetically modified mouse models (GEMMs) are essential to study the molecular mechanisms underlying PDAC progression and to evaluate potential therapeutic targets. In particular, transgenic preclinical mouse model Pdx1-Cre; LstopL-KrasG12D (KC) is currently considered the best model to mirror early human pancreatic pathology, as this mutation is found in 75%–95% of pancreatic cancers and in precancerous PanIN lesions (30). These mice develop the full spectrum of pancreatic tumor progression, from acinar to ductal metaplasia (ADM) and preneoplastic lesions (PanIN) to adenocarcinoma (31). Indeed, this mutation leads to the formation of PanIN similar to human PanIN in terms of activated signalling pathways and immune response establishment. Notably, it has been shown that Treg accumulate similarly in mouse and human PanIN (32). In addition, high Treg percentage in circulation and infiltrating predict poor prognosis in PDAC cancer patients (26).

Galectin-9 (LGALS9) is a 36-kDa tandem repeat galectin containing two carbohydrate recognition domains joined by a linker and which have a high affinity for β-galactoside residues. Galectin-9 has several reported receptors notably present in both immune cells and cancer cells with T-cell immunoglobulin and mucin-domain containing-3 (TIM-3) being the most important and described one (33). The Gal-9/TIM-3 pathway is functional in a wide range of human cancer cells (34), and Gal-9 expression is essentially associated with poor prognostic in many cancers such as renal cell carcinoma or in acute myeloid leukaemia, gastric carcinoma, and pancreatic ductal adenocarcinoma (35, 36). Recently, LGALS9, which is highly expressed by PDAC tumor cells, has emerged as a promising new biomarker in PDAC and a target for immunotherapy. More precisely, among multiple solid tumors, PDAC had the highest *LGALS9* expression, and galectin-9 mRNA levels were much higher than those of PD-L1 (37). In PDAC, LGALS9 has been described as a major actor of the immunosuppressive microenvironment regulation through type 2 macrophages (M2) and immunosuppressive γδ T cells (23, 24, 38–40). In this context, given the major role of LGALS9 in Treg immunosuppressive activity, we propose to evaluate our anti-LGALS9 immunotherapy protocol, specifically developed to neutralize LGALS9 and Treg-suppressive activity, in a transgenic KC mouse model of pancreatic cancer. Our primary aims are to (i) assess the expression of LGALS9 and Tregs in KC mice, (ii) validate the use of a human-specific anti-Galectin-9 on murine Tregs, and subsequently (iii) analyze its influence on LGALS9 expression, Treg infiltration, and, lastly, (iv) PaniN progression in anti-galectin-9-treated KC mice.

## Materials and methods

### Pdx-1-Cre; LStopL-KrasG12D mouse model of early pancreatic carcinogenesis

Pdx1-Cre mice were obtained from the Mouse Models of Human Cancer Consortium (MMHCC, Frederick, MD, USA). LStopL-KrasG12D mice were obtained from Dr I Van Seuningen (Univ. Lille, Inserm, CHU Lille, UMR 9020—CANTHER, Lille, France) (41). All procedures were in accordance with the guidelines of the Animal Care Committee (Comité Ethique Expérimentation Animale Nord Pas-de-Calais, #AF042008 and #00422.02). LSL-KrasG12D and Pdx1-Cre mice were maintained as heterozygous lines and crossed to obtain KC mice (Pdx1-Cre; LSL-KrasG12D) and corresponding WT controls (Pdx1-Cre; KrasWT). Tail snips, harvested from offsprings of mice, were digested overnight, and genomic DNA was extracted using the Nucleospin Tissue kit (Macherey Nagel, Hoerdt, Germany) according to the manufacturer’s instructions. The Cre and KrasG12D alleles were identified by PCR as already described elsewhere (41). Pancreas were isolated from WT or KC mice, and then, the head, the body, and the tail of the pancreas were split into three parts. Each part was placed in TRIzol (Thermo Fisher Scientific, Waltham, MA, USA) for RNA extraction or in 1 ml of RPMI (Thermo Fisher Scientific, Waltham, MA, USA) for enzymatic digestion to flow cytometry analysis and formalin-fixed paraffin-embedded for IHC analysis.

### KC mice (Pdx1-Cre; LSL-KrasG12D) and WT controls (Pdx1-Cre; KrasWT) anti-galectin-9 treatment

Two-month-old KC and WT mice were treated once a week with subcutaneous injection of anti-LGALS9 mAb (1g3, 20 µg) (Biotem, Apprieu, France) or isotype mouse IgG1K (20µg) (Biolegend, San Diego, CA, USA) immunotherapy for 8 weeks. Mice were bled after 1 week, 4 weeks, and at euthanasia for cytometry analysis (**Supplementary Figure S1**).

### Pancreas and spleen dissociation

Murine splenocytes and pancreatic cells were obtained under sterile conditions from mice euthanized by cervical dislocation (approval number CEEA 152010). The spleens and pancreas were collected and placed in MACS buffer (Miltenyi, Gladbach, Germany). They were then dissociated under sterile condition using gentleMACS™ M Tubes for the spleen and the Tumour Dissociation Kit and gentleMACS™ M Tubes (Miltenyi, Gladbach, Germany) for the pancreas using the gentleMACS™ Octo Dissociator (Miltenyi, Gladbach, Germany) following the manufacturer’s instructions. The final suspension was passed through a 100-μm cell strainer (Sigma Aldrich, St. Louis, MO, USA) into 1× Phosphate Buffer Saline (PBS) without Ca^2+^/Mg^2+^ (PBS−/−) and kept at 4°C until further use.

### Murine Treg and Tconv isolation

CD4+CD25+ (Treg) or CD4+CD25− conventional T cells (Tconv) cells were obtained from dissociated spleen, thanks to the murine CD4+CD25+ Regulatory T Cell Isolation Kit according to the supplier’s instructions (Miltenyi, Gladbach, Germany). Staining for cytometry control was done following the procedure below using the panel described in **Table 1**.

**Table 1:** Antibodies for cytometry analysis on C57Bl/6 mice model.

Antibody	Clone	Isotype	Clone
CD4-FITC	GK1.5	Rat IgG2b-FITC	ES265E12.4
CD25-PE	4E3	Rat IgM-PE	ES2613D3.4
Anti-FOXP3-APC	3G3	Mouse IgG1-APC	IS5-21F5

### Murine Treg and Tconv culture

Tconv cultures and Tconv/Treg co-cultures were done in RPMI 1640 medium supplemented with 1mM sodium pyruvate, 1× MEM Non-essential Amino Acids Solution, 25mM HEPES, 50μM 2-mercaptoethanol, 10μg/ml gentamicin (Thermo Fisher Scientific, Waltham, MA, USA), and 10% v/v foetal calf serum (Thermo Fisher Scientific, Waltham, MA, USA). Each proliferation assay was carried out in triplicates and estimated in count per minute (cpm), and results were normalized compared with non-treated condition.

### Flow cytometry

Single-cell suspension from the blood, spleen, and pancreas dissociation was washed with 1× PBS (Thermo Fisher Scientific, Waltham, MA, USA) and treated with mouse FcR blocking reagent for 15 min at RT and labelled with fluorochrome-conjugated antibodies according to the manufacturer’s instructions. mAb anti-CD45, CD4, CD25, and CD127 (Miltenyi, Gladbach, Germany) were used for cell surface staining with the appropriate isotypic controls (**Table 2**). Intracellular staining was achieved with an intracellular staining kit according to the manufacturer’s instructions for the use of anti-FoxP3 and anti-galectin-9 mAb (**Table 2**) (Miltenyi, Gladbach, Germany). The labelled cells were filled up with 300 μL of PBS−/−, and the fluorescence was analyzed by flow cytometry at the BD FACS Canto II™ (Becton Dickinson, Franklin Lakes, USA). The cytometry results were then analyzed with FlowJo™ v10.6.2 software (Becton Dickinson, Franklin Lakes, USA). The results were expressed as an MFI ratio (RFI).

**Table 2:** Antibodies for cytometry analysis in KC (Pdx1-Cre;LSL-KrasG12D) and corresponding WT controls (Pdx1-Cre;KrasWT) mice.

Antibody	Clone	Isotype	Clone
CD4-APC-Vio770	REA604	REA Control -APC-Vio770	REA293
CD25-VioBright FITC	REA568	REA Control -VioBright FITC	REA293
Anti-Galectin-9-APC	RG9-35	Rat IgG2a-APC	ES26-15B7.3
Anti-FoxP3-PE, human and mouse	3G3	Mouse IgG1-PE	IS5-21F5
CD127-PE-Vio770	A7R 34	Rat IgG2a-PE-Vio770	ES26-15B7.3
CD45 percp Cy5.5	30-F11	IgG2b percp Cy5.5	A95-1

### Immunohistochemistry and PanIN scoring

Pancreas were fixed in 4% (w/v) buffered formaldehyde, embedded in paraffin, cut at 4-µm thickness, and applied on SuperFrost® slides (Menzel-Glaser, Braunschweig, Germany). Slides were deparaffinized using a series of xylol–ethanol baths. Endogenous peroxidase activity was inactivated by H_2_O_2_ (1.5%, v/v, 30 min) followed by antigen retrieval Dako Real citrate buffer (microwave, 700 W; 20 min). Thereafter, sections were incubated with 5% (v/v) goat serum for 30 min to reduce non-specific binding. The antibodies were used as followed: anti-LGALS9 (1:200, # ab69630) and anti-CD8 (1/1000, #ab237723). Sections were incubated for 1 h with biotinylated rabbit IgG (Vector Laboratories, Peterborough, UK) followed by a 1-h incubation with ABC/PO complex Vectastain Elite Kit (Vector Laboratories, Peterborough, UK). All the slides were prepared extemporaneously with 4 min and 30 s of staining for anti-galectin-9 slide and 5 min for anti-CD8 slides. Slides were then scanned with the Axioscan Z1 from BICelL Platform (nom du responsable, University of Lille). Haematoxylin–eosin–saffron (HES) staining was performed on formalin-fixed tissue. The scoring of PanIN surface was manually performed using a Macro specifically developed on FIJI software for this application (42). CD8 scoring was achieved using FIJI software v1.0 by dividing the number of CD8+ cells by the surface area of the tissue sample in square millimetre.

### RNA extraction

Total RNA from Peripheral Blood Mononuclear Cells (PBMC) and pancreas were extracted using the TRIzol reagent (Thermo Fisher Waltham, MA, USA) method according to the manufacturer’s instructions. RNA concentration and purity were measured by spectrophotometric methods (Ultrospec 3000, Pharmacia Biotec). Total RNA were stored at −80°C until further use.

### Retro-transcription and quantitative PCR

The Superscript™ II Transcriptase Reverse Kit was used for retro-transcription (RT) (Invitrogen, Carlsbad, USA). RT was performed using 1 µg of total RNA. The RT-Q-PCR reactions were performed for selected genes (**Table 3**), according to the manufacturer’s instructions. Briefly, we used the 2× MESA GREEN qPCR MasterMix Plus for SYBR 258 Assay (Eurogentech, Liège, Belgique) in a 96-well qPCR plate (Sarstedt, Nümbrecht, Germany) covered by an optical seal (Dutcher, Brumath, France). The Mx3005P™ thermocycler and sequence detection system (Agilent Technologies, Santa Clara, USA) was used for amplification and analysis. In each reaction, 10 ng of reverse transcribed RNA (based on initial RNA concentration) was used. All primers were used at 400 nM in a 20-µl reaction. Quantitative analysis was performed based on the cycle threshold (Ct) value for each well and calculated using MxProSoftware version 4.10 (Agilent Technologies, Santa Clara, USA). The results were normalized using three housekeeping (HKG) genes, namely, 18S, GAPDH, and HPRT, and data were represented as fold differences by the 2^−ΔΔCt^ method, where ΔCt = Ct target gene − Ct HKG.

**Table 3:** Primers for RT-Q-PCR analysis.

Gene Name	Forward Primers	Reverse Primers
*LGALS-9*	ACTTTCAGAACAGCTTCAATGGA	AGTCCATCATGATATCAGGCAAT
*18S*	TCAAGAACGAAAGTCGGAGG	GGACATCTAAGGGCATCACA
*HPRT1*	CCCTGGCGTCGTGATTAG	ATGGCCTCCCATCTCCTT
*GAPDH*	CCATCAATGACCCCTTCATTG	CTTGACGGTGCCATGGAATT

### Analysis of galectin-9 transcripts from datasets

The relative expression of Gal-9 mRNA in pancreatic samples from six KrasG12D (KC) and five wild-type WT mice from the GSE53659 mouse cohort (https://www.ncbi.nlm.nih.gov/geo/query/acc.cgi?acc=GSE53659) was performed using the Gene Expression Omnibus (GEO2R) analysis tool (https://www.ncbi.nlm.nih.gov/geo/geo2r/).

### Immunofluorescence

A total of 10^4^ cells (Tconv or Tregs) were treated with mouse FcR blocking reagent for 15 min at room temperature (Miltenyi, Gladbach, Germany) and then fixed with 4% (v/v) paraformaldehyde in 1× PBS (Santa Cruz Biotechnology, USA) before being plated on slides by centrifugation at 800 rpm for 5 min (Cytospin 4 ThermoShandon, France). Cells were then washed three times with 1× PBS (Thermo Fisher Scientific, Waltham, MA, USA) and permeabilized with 0.5% (v/v) Triton X-100 (Sigma Aldrich, St. Louis, MO, USA) in 1× PBS. Following three washing steps, blocking was done with a solution made of 1× PBS completed with bovine serum albumin (BSA) 2% (v/v) (Sigma Aldrich, St. Louis, MO, USA) and 0.3% (v/v) Triton X-100. Both primary and secondary antibodies incubations were carried out in 1× PBS containing 0.5% (v/v) BSA, 2 h at room temperature. The anti-galectin-9, 9CT clone, was used as primary Ab at 4 μg/ml with a goat anti-rabbit IgG (H+L) coupled to Alexa Fluor® 555 as secondary at 8 μg/ml (Thermo Fisher Scientific, Waltham, MA, USA). Nuclei were stained with 4′6′-diamidino-2- phenylindole (Sigma Aldrich, St. Louis, MO, USA) for 5 min at room temperature. Slide and blade assembly was done with Mowiol® 4-88 (Sigma Aldrich, St. Louis, MO, USA), and the slides were kept in the dark at 4°C until further use. A control was done without primary Ab to eliminate background noise due to non-specific bindings of the secondary Ab. Slide analysis was done using a LSM780 confocal microscope (Carl Zaiss, Oberkochen, Allemagne) under Zen software (Carl Zaiss, Oberkochen, Allemagne).

### Mouse model for anti-Galectin-9 clone 1G3 assessment in murine models

In accordance with institutional guidelines, C57BL/6 (Charles River, Ecully, France) were housed under specific pathogen-free conditions at the animal facility of the Lille Pasteur Institute (PLEHTA, Lille, France) until further use. Spleens were recovered; T-cell isolation and consecutive assays were achieved according to protocols described above and below.

### Proliferation assays

The suppressive activity of murine galectin-9 or murine Treg was measured by their ability to inhibit the proliferative response of autologous murine leucocytes alone or in a mix leucocyte reaction (MLR), respectively. A total of 10^5^ CD25− were cultured alone, with recombinant murine galectin-9 (rLGALS9) (R&D Systems, Minneapolis, MN, USA) at 3 μg/ml or with autologous Tregs (50,000) for 72 h in round bottom 96-well plates. Cells were activated with plate-bound anti-CD3 (5 μg/ml) mAb (Miltenyi, Gladbach, Germany), incubated at 37°C for 2 h before the culture and soluble anti-mouse CD28 functional Grade Purified (5 μg/ml) (Clinisciences, MontRouge, France) added at the time of the culture. When needed, murine rLGALS9 or Tregs were pre-incubated with anti-LGALS9 mAb (clone 1G3, Biotem, Apprieu, France) or its IgG1 Kappa isotypic control (Biolegend, San Diego, CA, USA) in the necessary medium for at least 2 h before adding to the culture without washing. Proliferation was measured after [3H] thymidine (1 μCi/well) (PerkinElmer, Courtaboeuf, France) incubation for the last 18 h before harvesting. Radioactivity incorporation was determined using a β-counter (1450 Trilux, Wallac, Finland).

### Statistical analysis

Data were analyzed and represented using the statistical package GraphPad Prism 8.0.0 for Windows (GraphPad Software, Inc., San Diego, CA). All quoted p-values are two-sided, with p ≤ 0.05 (*), p ≤ 0.01 (**), p ≤ 0.001 (***), and p ≤ 0.0001 (****) being considered statistically significant for the first and highly significant for the others. The Mann–Whitney test was used after testing the non-Gaussian distribution of our values with Shapiro–Wilk normality test.

## Results

### Expression of galectin-9 in the pancreas of KC mice

**Figure 1:**
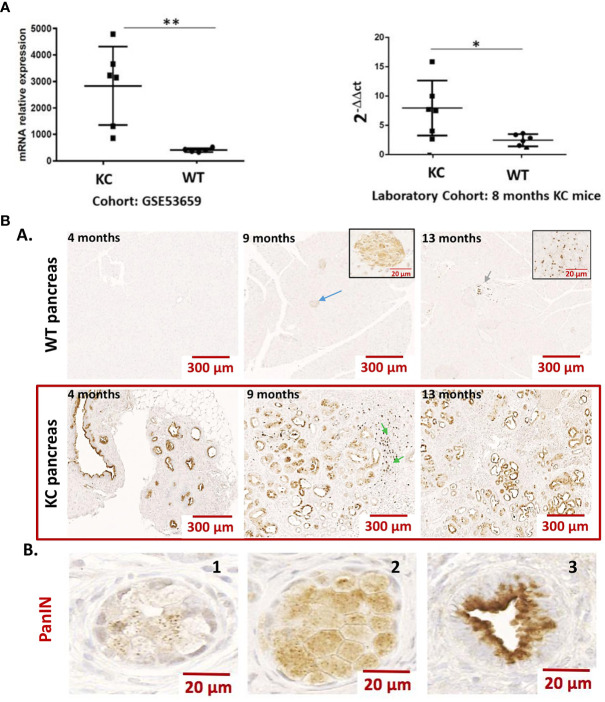
**(A)** Expression of Gal-9 transcripts in the pancreas of WT vs. KC mice. Gal-9 mRNA relative expression from pancreatic samples of Kras^G12D^ (KC) and WT mice using cohort (GSE53659) from Gene Expression Omnibus (n= 6 for KC and n=5 for WT) and from an in-house laboratory cohort (n= 6 for KC and n=6 for WT). All quoted p-values are two-sided. p-values from Mann–Whitney with p ≤ 0.05 (*) and p ≤ 0.01 (**). **(B)** Representative expression of LGALS9 in the pancreas of WT vs. KC mice. **(A)** Anti-galectin-9 immunohistochemistry on FFPE sections of WT and KC mouse pancreas, collected at 5, 9, and 13 months. Gal-9 was absent or minimally expressed in WT mice. The blue arrow in the 9-month WT mice shows a staining in the Langherans islets. The green arrows at WT 13 months and KC 9 months are an example of staining on a leucocyte infiltrate. The staining in these structures is enlarged in the inserts in 9- and 13-month WT mice pictures. In the pancreas of KC mice, all PanIN have an important LGalS9 labeling. The green arrows at 9 months in KC mice shows lymphocyte infiltration in nearby PanIN. **(B)** Example of LGALS9 staining during ADM (B1 and B2) and in PanIN (B3). The staining is found in the cytoplasm of both ADM and PanIN. In PanIN (B3), there is a translocation from the nucleus to the basal pole and a high-intensity staining at the apical pole.

We analyzed the expression of *LGALS9* transcripts in the pancreas of KrasG12D (KC) versus WT mice (GSE53659 dataset) (26) using bioinformatics from the public Gene Expression Omnibus (GEO). The analysis shows a statistically significant difference in relative transcript expression in KC, which had more than five times more transcripts than the normal WT control. Using this bioinformatics approach, we were able to confirm the results of our internal comparison cohort, which showed a statistically higher level of relative *galectin-9* expression in KC mice compared to the WT control (**Figure 1A**).

**Figure 2:**
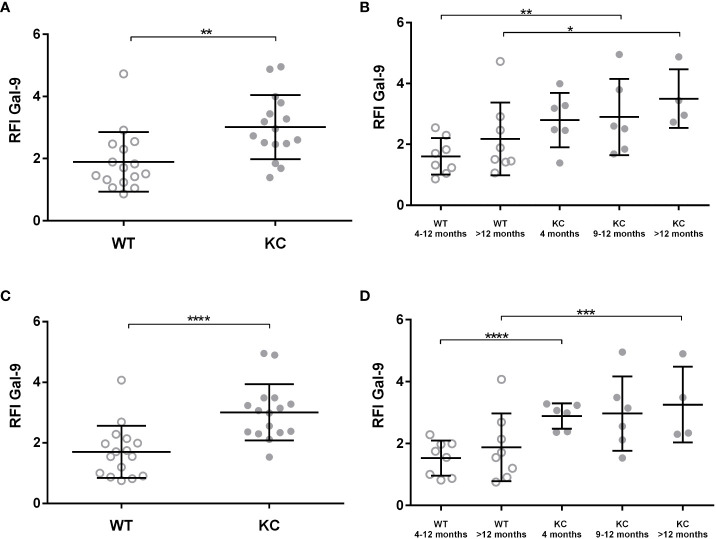
Expression of LGALS9 in the pancreas of WT versus KC mice. LGALS9 expression was analyzed by cytometry and is given is RFI in relation to the control isotype. Pancreases were stained after mechanical and enzymatic digestion. LGALS9 expression was analyzed directly after permeabilization and staining of total pancreatic cells lysate. **(A,B)** Overall expression of LGALS9 in the pancreatic lysate. **(B)** LGALS9 expression in the different age subgroups. **(C)** Expression of LGALS9 in pancreatic cells (CD45−) after leucocyte exclusion (CD45+) of total pancreatic lysate. **(D)** Detailed representation of LGALS9 expression in pancreatic cells (CD45−) only by separating the different age subgroups. Error bars represent standard deviations. All quoted p-values are two-sided. p-values from Mann–Whitney with p ≤ 0.05 (*), p ≤ 0.01 (**), p ≤ 0.001 (***), and p < 0.0001 (****).

**Figure 3:**
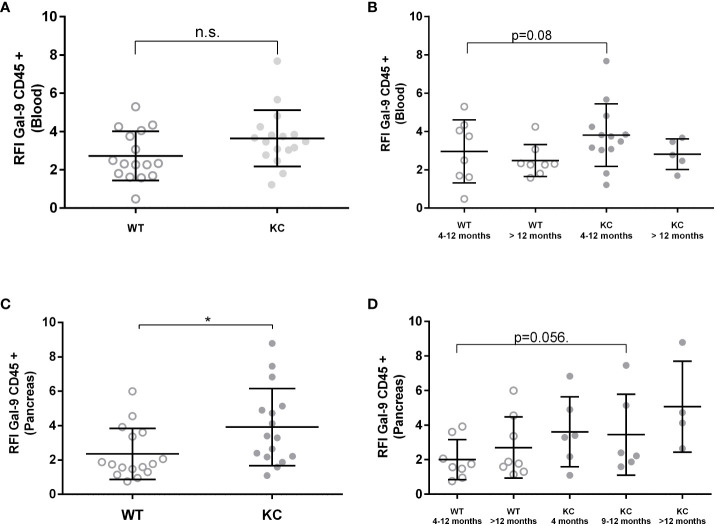
Expression of Gal-9 by infiltrating and peripheral immune cells in WT or KC mice. LGALS9 expression was analyzed in both peripheral **(A, B)** and in pancreatic infiltrating leucocytes **(C, D)**. Peripheral leucocytes were stained from peripheral blood after red blood cell lysis. **(B)** LGALS9 expression in peripheral leucocytes in the different age subgroups. **(D)** LGALS9 expression in infiltrating leucocytes in the different age subgroups. Error bars represent standard deviations. All quoted p-values are two-sided. p-values from Mann–Whitney with p≤ 0.05 (*). n.s. is for not significative.

LGALS9 expression was then examined by immunohistochemistry in formalin-fixed paraffin-embedded pancreas from WT and KC mice at 4, 9, and 13 months of age. LGALS9 was absent or weakly expressed in WT mice. In WT-positive mice, LGALS9 staining was weak and localized to the cytoplasm and nucleus of pancreatic cells. LGALS9 expression in KC mice was essentially and strongly restricted to the PanIN at all stages (**Figures 1B**, A). Strong LGALS9 staining was observed at the apex and in the nucleus. A more diffuse and low-intensity label was also observed in the cytoplasm (**Figures 1B**, A). LGALS9 staining is already present in the initial acinar to ductal reprogramming metaplasia (ADM) (**Figures 1B**, B,1–2) and was further enhanced in PanIN (**Figures 1B**, B,3). Finally, we also found expression in Langerhans islets (**Figures 1B**, A, blue arrow), mononuclear cells (**Figures 1B**, A, green arrow) in the vasculature of both KC and WT mice.

In addition, we analyzed LGALS9 cellular expression within the pancreas tissue by cytometry directly after pancreas digestion. We determined the overall expression of LGALS9 in total pancreas (**Figures 2A, B**) and in sorted non-immune pancreatic cells (CD45−) (**Figures 2C, D**). There was a significant increase (RFI: 1.04) in the overall expression of LGALS9 in the pancreas (CD45−; CD45+) and also in the pancreatic cells (CD45−) of KC (RFI: 3.03) versus WT (RFI: 1.63) mice (**Figures 2A, C**). This increase was even found and significant for all age subgroups (**Figures 2B, D**).

LGALS9 protein expression was also analyzed in both circulating and infiltrating leucocyte (CD45+) (**Figure 3**). Analysis of LGALS9 expression in peripheral immune cells did not show any significant difference between KC (RFI: 3.44 ± 0.34) versus WT (RFI: 2.86 ± 0.35) mice (**Figure 3A**). Although no other statistically significant differences could be found, we observed a trend showing that LGALS9 expression in peripheral immune cells decreased with age in KC (RFI: 3.91) and WT (RFI: 2.35) mice (p~0.08) (**Figure 3B**). Finally, the analysis of LGALS9 protein expression in pancreatic infiltrating immune cells (CD45+) showed a significant increase in LGALS9 expression in KC compared to WT mice (**Figure 3C**). However, this difference was not statistically significant when comparing subgroups (**Figure 3D**). Note that the p-value between WT 4–12 months and KC 4–12 months is 0.056 in (**Figure 3D**), indicating a tendency for LGLS9-positive immune cells to infiltrate KC mice.

### T CD4+ and Treg prevalence in the KC model

**Figure 4:**
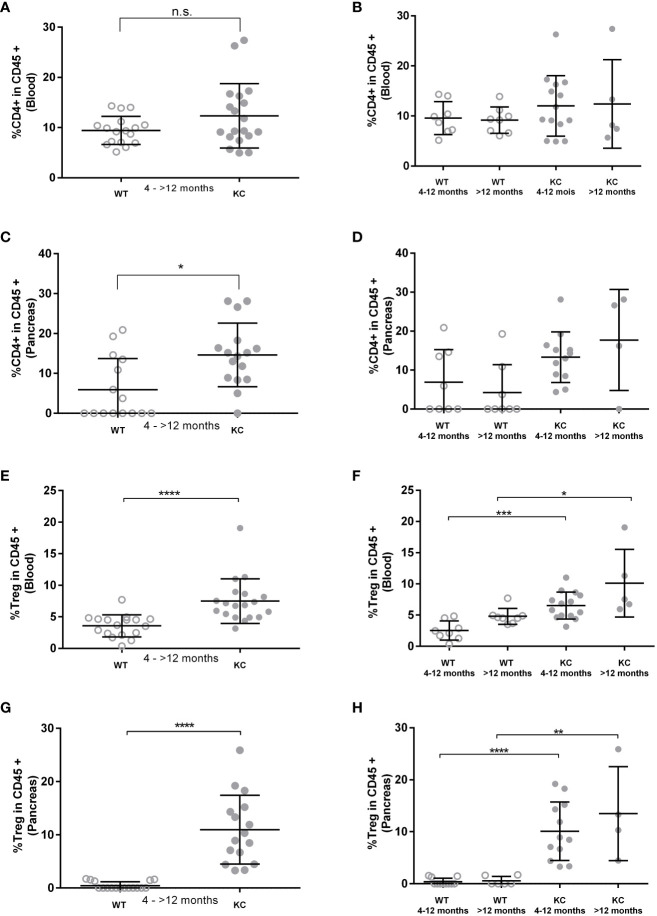
Evaluation of TCD4+ and Treg rates in peripheral and pancreatic-infiltrating immune cells. The circulating and infiltrating rates of TCD4+ **(A–D)** and Treg **(E–H)** cells was determined by cytometry based on the expression of CD45+ CD4+ and CD45+ CD4+, Foxp3+, and CD127−/low, respectively. Error bars represent standard deviations. All quoted p-values are two-sided p-values from Mann–Whitney with p ≤ 0.05 (*), p ≤ 0.01 (**), p ≤ 0.001 (***), and p ≤ 0.0001 (****). n.s. is for not significative.

Peripheral TCD4+ and Treg rates and infiltrating Tconv and Treg rates were analyzed by flow cytometry analysis in blood and pancreatic lysate, respectively. We found no significant differences in peripheral TCD4+ levels between KC and WT mice (**Figure 4A**). However, a significant increase was observed in pancreatic infiltrating TCD4+ levels between KC and WT mice (**Figure 4C**). However, no significant difference was observed when comparing subgroups (**Figures 4B, D**). Moreover, one can observe that more than 50% of the WT mice had no lymphocyte infiltration, whereas all KC mice had some (**Figures 4C, D**). Analysis of Treg levels showed a significant increase in both circulating (**Figures 4E, F**) and infiltrating (**Figures 4G, H**) compartment in KC mice compared to WT mice of the same age. Finally, there was an increase in both peripheral and infiltrating Treg levels regardless of age, indicating Treg circulation and infiltration as an early and sustain event of development.

### Validation of 1G3 for use in a murine model

**Figure 5:**
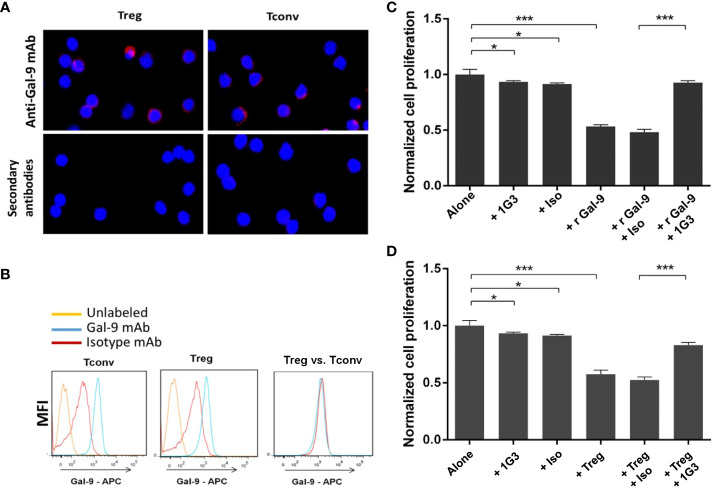
Validation of anti-galectin-9 (1G3 clone) for use in a murine model. LGALS9 (Gal-9) protein expression in murine Tconv and Treg was studied by IF **(A)** and flow cytometry **(B)**. **(C)** Blocking of the immunosuppressive recombinant murine galectin-9 (r Gal-9) by 1G3 in lymphocyte proliferation assays. Tconv cultured for 72 h alone or with r Gal-9 in TCR stimulating conditions with or without the anti-Gal-9 antibody or the isotypic control (IgG1K, Iso). **(D)** Blocking of the immunosuppressive ability of Tregs by 1G3 (anti Gal-9) in MLR assays. Tconv cultured for 72 h alone or with autologous Tregs (ratio 2:1) in TCR-stimulating conditions with or without the anti-Gal-9 antibody or the isotypic control (IgG1K, Iso). Error bars represent standard deviations. All quoted p-values are two-sided p-values from Student’s t-test with p ≤ 0.05 (*), and p ≤ 0.001 (***).

Since we developed an anti-LGLS9 for human application, and given the sequence homology of both the human and murine form of LGALS9, we took the chance to use this antibody in murine models. We therefore investigated the ability of anti-LGALS9 (1G3) to neutralize murine galectin-9 and murine Treg (**Figure 5**).

First, we evaluated the intracellular expression of LGALS9 by immunofluorescence in murine Treg and Tconv from C57BL/6 WT mice. We found intracellular expression of murine LGALS9 in both Treg and Tconv (**Figure 5A**). Then, we confirmed the intracellular expression of LGALS9 by flow cytometry and observed a similar expression level of LGALS9 between freshly sorted Tconv and Treg (**Figure 5B**).

In order to evaluate whether the anti-LGALS9 (1G3) antibody was able to neutralize murine LGALS9, we tested its ability to inhibit the immunosuppressive potential of rLGALS9 by proliferation assays on murine Tconv. As expected, rLGALS9 efficiently inhibited the proliferation of activated Tconv (47± 1.5%) in a statistically significant manner (p<0.001). The addition of anti-LGALS9 restores the proliferation of Tconv (92± 1.8%) and thus significantly inhibits the effect of rLGALS9 (p<0.001) (**Figure 5C**). No immune restoration was observed with the isotypic control (IgG1). In addition, there was a slight decrease in Tconv proliferation that was statistically significant (p<0.05) between 1G3 mAb (7.7 ± 1%) and its isotypic control (9.9± 3%).

To assess whether the anti-LGALS9 (1G3) antibody was also able to neutralize murine Tregs, we tested its ability to inhibit the immunosuppressive potential of Tregs using mixed lymphocyte reaction (MLR) assays performed as suppression assays on murine Tconv (**Figure 5D**). Activated Tconv proliferate *in vitro* and, as expected, co-culture of Tconv with autologous Treg (ratio 2:1) showed that autologous Treg induced a decrease (43± 3.6%) in the proliferation of activated Tconv. MLR suppression test confirmed that splenic murine Treg isolated *ex vivo* and under activation conditions possessed immunosuppressive activity. The addition of anti-LGALS9 to this co-culture partially restored proliferation (83 ± 2.3%), indicating inhibition of the suppressive function of Treg for which LGALS9 is partly responsible.

### *In vivo* evaluation of the 1G3 anti-galectin-9 antibody efficacy in a cohort of KC mice

Given the long latency characteristic of the model, and the early infiltration of Tregs, we decided to evaluate treatment in 2-month-old mice. A total of 12 mice were divided into different groups and treated with or without anti-LGALS9 mAb for 2 months (1 injection per week for 8 weeks, **Supplementary Figure S1**).

### Impact of 1G3 antibody on LGALS9 expression in KC mice

#### Impact of 1G3 on LGALS9 expression in the pancreas of KC mice

**Figure 6:**
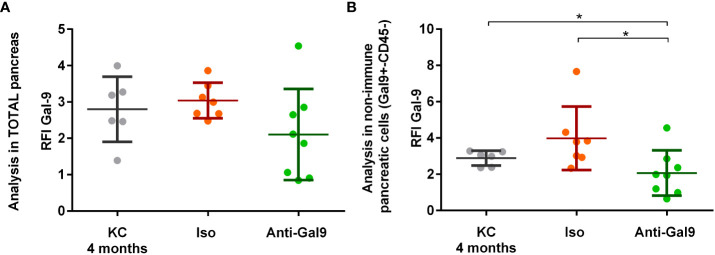
Impact of 1G3 anti-galectin-9 on Gal-9 expression in the pancreas of KC mice. Two-month KC mice were treated weekly with 10 μg of anti-gal-9 or isotype (Iso) for 2 months. Gal-9 expression was analyzed by cytometry and is given in RFI compared to the isotype. **(A)** Overall expression of Gal-9 in the pancreatic lysate after digestion from KC mice receiving no treatment, IgG1k isotype (Iso), or 1G3 anti gal-9. **(B)** Analysis of the Gal-9 expression in non-immune pancreatic cells (CD45−) obtained after exclusion of leucocyte (CD45+) from pancreatic lysate. p-values from Mann–Whitney. All quoted p-values are two-sided, with p≤ 0.05 (*).

Mice have been treated for 2 months with 10 μg of 1G3 anti-galectin-9 or isotypic control IgG1 kappa weekly. We observed a decrease in the overall LGALS9 expression in the pancreas of mice treated with anti-LGALS9 (RFI: 2.06) versus isotype (RFI: 3.04) and between isotype versus non-treated mice (RFI: 3.01) (**Figure 6A**). The difference was nearly significant (p= 0.056) between mice who received isotype or anti-galectin-9. In addition, we observed a significant decrease in the expression of LGALS9 by pancreatic cells (CD45−) in mice treated with anti-LGALS9 (RFI: 2.06) versus isotype (RFI: 3.98) or nothing (RFI: 3.01) (**Figure 6B**).

#### Impact of 1G3 on LGALS9 expression in peripheral and infiltrating immune cells (CD45+)

**Figure 7:**
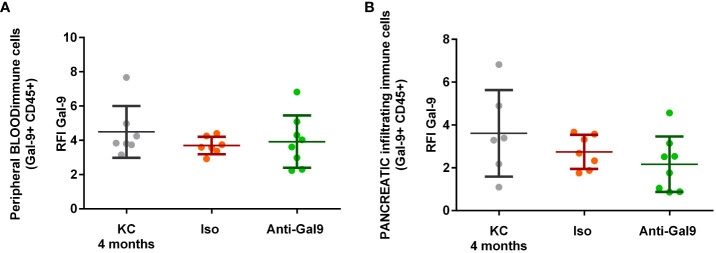
Impact of 1G3 anti-Gal-9 mAb on Gal-9 expression in peripheral and infiltrating immune cells (CD45+) of KC mice. **(A)** Leucocytes from peripheral blood after red blood cells lysis were stained and analyzed by flow cytometry. **(B)** Infiltrating leucocytes were stained after mechanical and enzymatic digestion of the mice pancreas and analyzed by flow cytometry. Iso: IgG1K isotypic control of the anti-galectin-9 (Anti-Gal9) 1G3 mAb.

No differences in the expression of LGALS9 by infiltrating or peripheral immune cells (CD45+) were observed (**Figures 7A, B**). There is no significant difference in LGALS9 expression among peripheral leucocytes (**Figure 7A**) from mice receiving no treatment (RFI: 4.49), isotype (RFI: 3.69), or anti-galectin-9 (3.92). There is no significant difference in LGALS9 expression among pancreas-infiltrating leucocytes (**Figure 7B**) from mice receiving no treatment (RFI: 3.35), isotype (RFI: 2.74), or anti gal-9 (RFI: 2.17) despite a small decrease.

### Impact of 1G3 on CD4+ effector T cells and Tregs in peripheral blood and pancreas of KC mice

**Figure 8:**
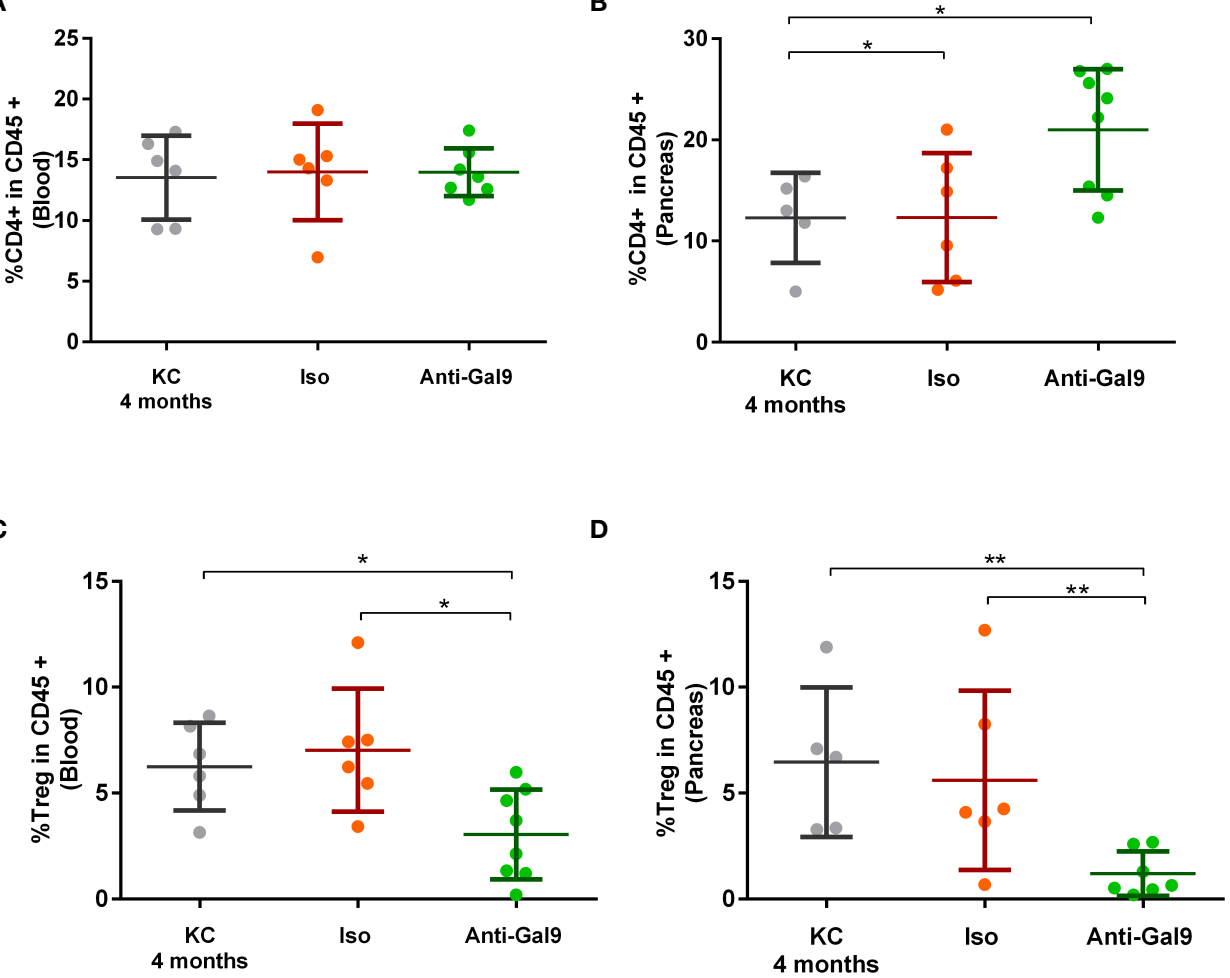
Impact of 1G3 anti-galectin-9 mAb on effector and regulatory T cells in KC mice. The circulating (A,C) and infiltrating (B,D) rates of TCD4+ **(A, B)** and Treg cells **(C, D)** was determined by cytometry based on the expression of CD45+ CD4+ for effector CD4+ T cells and CD45+ CD4+, Foxp3+. and CD127−/low for Tregs. Error bars represent standard deviations. p-values from Mann–Whitney. All quoted p-values are two-sided, with p≤ 0.05 (*) and p≤ 0.01 (**).

We observed no difference in CD4+T prevalence at the peripheral level between non-treated (13.54%), isotype (13.99%), or 1G3 anti-galectin-9 mAb (13.97%)-treated mice (**Figure 8A**). On the other hand, we observed a significant increase in TCD4+ levels infiltrating the pancreas following treatment with 1G3 (20.1%) versus isotype treated (12.33%) or untreated (12.28%) (**Figure 8B**). Analysis of Treg rates showed a high reduction in peripheral and infiltrating Treg levels after 1G3 anti-galectin-9 treatment, respectively (**Figures 8C, D**), indicating an increase in CD4+/Treg ratio in favour of the effector response.

### Impact of 1G3 anti-galectin-9 on PanIN progression

**Figure 9:**
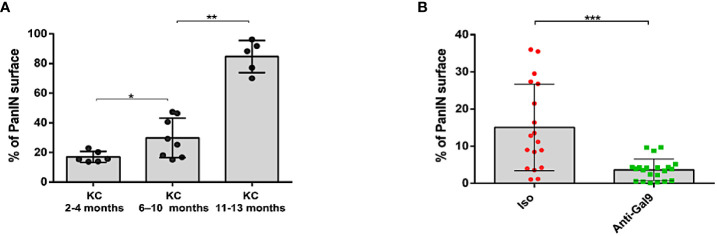
Study of PanIN surface in pancreas of KC mice. The scoring of PanIN surface was manually performed using a Macro specifically developed on FIJI software for this application. **(A)** Total PanIN surface area increasing with age of the mice. **(B)** Decrease in total PanIN surface area in mice treated with anti-Gal-9. Each plot corresponds to one slide; three slides were analyzed per FFPE sample. Error bars represent standard deviations. p-values from Mann–Whitney. All quoted p-values are two-sided, with p≤ 0.05 (*), p≤ 0.01 (**), and p≤ 0.001 (***).

In order to determine whether the decrease in LGALS9 and Treg levels could be correlated with the evolution of the disease, we measured the total surface area of the PanIN on pancreatic sections. We first validated our scoring by showing a statistically significant increase in the total surface area of PanIN proportionally to the evolution of the pathology and the age of the mice (**Figure 9A**). Finally, we observed a decrease in the total surface area of PanIN in mice treated with 1G3 (**Figure 9B**). All untreated mice presented ADM and PanIN 1. Five of six untreated mice presented at least one PanIN Ib/II. All treated mice presented ADM or PanIN1, and one mouse harboured one PanIN Ib/II.

## Discussion

Currently, PDAC patients fail to respond to immunotherapy and this failure can be attributed to highly immunosuppressive immune cells including tumor-associated macrophages, MDSCs, γδ T cells, and Tregs found in the tumor microenvironment (23, 24, 38, 43). Recently, several publications demonstrated the importance of galectin-9-mediated immunosuppressive infiltrates that populate PDAC lesions especially M2 and γδ T cells (23, 25). Interestingly, it has recently been reported that among multiple solid tumors PDAC had the highest galectin-9 expression, and galectin-9 mRNA levels were much higher than those of PD-L1 (37). This is particularly interesting in view of the failure of anti PD-1 and PD-L1 in PDAC but also because galectin-9 has recently been described as a major resistance mechanism associated in anti-PD-1-resistant Kras tumors (44). Indeed galectin-9 overexpression has in the past few years emerged as a main adaptive mechanism of resistance to cytotoxic activity and immunotherapeutic approaches, mainly *via* its capacities to bind to other immunoregulatory molecules such as PD1 or PD-L1 alone or *via* its own receptors notably Tim-3 (45–48). This mechanism is also supported by its immunosuppressive effect on the TME. Indeed, the immunosuppressive properties of galectin-9 have been extensively described and can act on multiple immune cell subsets, notably by inducing apoptosis of T helper Th1 (49), inhibition of Th17 (50), expansion of immunoregulatory cells such as M2 macrophages (51), MDSC (52), and Treg (39, 50, 53) *via* the many receptors to which it binds (33). Here, we reported a significant increase in the overall expression of LGALS9 in the pancreatic lysate and in the pancreatic cells (CD45−) in KC versus WT mice aged between 4 and 13 months. Interestingly, our studies revealed that LGALS9 increase was observed very early in KC mice, as early as acinar to ductal metaplasia, which is the earliest preneoplastic lesion arising in KRAS-driven PDAC models. LGALS9 is then heavily expressed at the very early metaplastic stage in PanIN1, suggesting a key function in tumor progression. Moreover, other galectins, especially galectin-1 and galectin-3, have already been described as playing a major role in ADM and PDAC progression synergistically with RAS mutation (54, 55).

In parallel, we assessed the frequency of peripheral and tumor-associated CD4+ T cells and Treg in KC versus WT mice. We found no significant differences in both peripheral and infiltrating CD4+T cells levels. However, although no statistical difference was observed with respect to CD4+ T cell infiltration, we demonstrated that no lymphocyte infiltration could be seen in more than 50% of WT mice compared to KC mice. In addition, some KC mice had a high frequency of CD4+ T cells, which was never observed with WT mice. Finally, analysis of Treg prevalence showed their significant increase at both circulating and infiltrating sites for KC mice along with the age as young as 4 months. Additionally, a very low infiltration of CD8+ T lymphocytes was observed (**Supplementary Figure S2A**), which suggests the absence of an effective anti-tumor immunity until the inception of neoplasia. Our observations regarding Treg infiltration suggests their role right from the earliest step of the pre-invasive neoplastic process in the absence of an active anti-tumor immune response. These observations are in line with previous reports by Clark et al., where they described increased Treg levels without any CD8+ cells (56). Jang et al. employed an orthotopic model of KC pancreatic ductal epithelial cells to show that, in the context of PDAC, Treg plays a pro-tumoral role in conferring immunosuppressive properties to tumor-associated CD11c+ DCs. Finally, they demonstrated that genetic Treg cell depletion elicits an effective anti-tumor immunity dependent on CD8+ T-cell activation. Interestingly, they also showed that Treg cells accumulate around KC-PDAC grafts within 1 week, suggesting that the neoplastic cells themselves may play a direct role in promoting Treg cell infiltration (57). However, their study does not clearly explain the mechanisms by which Treg cells are recruited into the TME.

In this context, we decided to challenge KC mice with a neutralizing antibody, patented by our team, directed against LGALS9 (1G3 clone). Indeed, we have previously described that antagonizing only human LGALS9 with a specific monoclonal antibody is sufficient to both neutralize human Treg suppressive activity and induce a strong anti-tumor immune response in a humanized mouse model of NPC also known to express high level of LGALS9 (58). In addition, we previously validated the ability of the antibody to inhibit the effect of LGALS9 and thus restoring the proliferation of Tconv. Finally, we show that the anti-LGALS9 can restore proliferation of Tconv when co-cultured with Treg, indicating an inhibition of Treg suppressive function. The efficacy of 1G3 on human LGALS9 and murine LGALS9 can be explain by their strong homology. One possible explanation for this cross-effectiveness between the two species is that 1G3 targets the (TPAIPPMPHP) amino-acids (aa) sequence in human while targeting the (TPGIPPVYPTP) aa sequence for murine LGALS9, which shares 69% homologous. Unexpectedly, we observed a slight, but significant decrease in murine Tconv proliferation with 1G3, which was not observed with human Tconv. On the other hand, we did not find any statistically significant difference in proliferation with either 1G3 or its isotype, suggesting an aspecific toxicity. In addition, the efficacy of Gal-9 blockade was validated in a PDAC mouse model with established orthotopic KPC tumors, where it slowed tumor progression and prolonged mouse survival in a Dectin-1-dependent manner (23). We were therefore encouraged to test the capabilities of our antibody in an earlier model of PDAC development.

Considering the characteristics of the model, long latency along with fast increase in LGALS9 level and Treg prevalence between KC and WT mice as early as 4 months, we decided to evaluate the treatment on 2-month-old mice during 2 months.

The use of 1G3 led to a significant decrease in LGALS9 expression by pancreatic cells along with Treg prevalence at both the circulating and infiltrating level. This result can probably be attributed to the blockade of galectin-9/Tm-3 interaction. Indeed, both the galectin-9 and Tim-3 are expressed in solid tumors and act synergistically to counteract attack from host immune defence (33). According to current knowledge, when TIM3 is free of ligand, HLA− B-associated transcript 3 (BAT3), its main adapter, is retained at its cytoplasmic tail and helps in activating signalling of T cell (59). However, upon Gal9 binding, TIM3 oligomerise, and both Tyr256 and Tyr263, tyrosine at Tim-3 intracytoplasmic tail that allow BAT3 binding (60), are phosphorylated, which secondly triggers BAT3 release and unleash immunosuppressive function of TIM3 (49, 61). The interaction of galectin-9 and Tim-3 lead to immunosuppression either by directly inducing apoptosis of CD4+ or CD8+ effector activated T cells or indirectly by activating various immunoregulatory cells (Treg, M2, MDSC, etc.). Thus, blocking galectin-9/Tim-3 pathway, through anti-galectin-9 mAb, is expected to restore efficient immune response counterbalancing the suppressive TME. Nevertheless, Tim-3 has also been described as participating in the vicious circle of cancer development in both solid tumor and haematological malignancies (62–65), and the reduction in Tim-3 signalling could also lead to reduce tumor growth and aggressiveness. A better understanding of the double face of Tim3 is an important issue in many infectious or tumor diseases. Fortunately, data on its biology are accumulating and are likely to allow greater efficiency in the fight against these diseases (66). Interestingly, we observed a significant increase in infiltrating CD4+, but there was no evidence of a massive TCD8+ infiltration in KC mice after treatment with 1G3 (**Supplementary Figure S2B**). This highlighted the question about the establishment of an active anti-tumor immunity in this model. Indeed, several publications have suggested that Treg neutralization leads to CD8+ infiltration. However, this type of observation was obtained in old KC mice or in the more aggressive KPC model (57, 67). This is consistent with our data, since we only noticed CD8+ T-cell infiltration in the vicinity of high-grade PanIN (grade Ib or II) in untreated mice but also exceptionally in mice treated with 1G3. In this context, is the immune system able to recognize neoplastic lesions at the earliest signs of dysplasia (ADM, PanIN1), or do such lesions remain immunologically ignored until disruption of tissue architecture and danger signals? Our observations suggest the establishment of an immunosuppressive mechanism before an adaptive CD8+ T-cell response. Not only LGALS9 is implicated in high Treg ratio, as it has been demonstrated using tumor-derived KC mice, but also as an important chemokine production like CXCl16 and CXCR2 (68, 69). Indeed, this hypothesis has already been raised by Clark et al. who showed that Treg infiltrate very early in the disease progression, at the preinvasive state and before experimental evidence of an adaptive CD8+ T-cell response, suggesting that Treg infiltration does not necessarily represent only the contraction phase of an earlier immune response. In fact, their data suggest that CD8+ T-cell responses encounter multiple pre-existing components of host immunosuppression and are therefore either blunted before immunologic elaboration or never initiated at all at the very early stage. Finally, we believe that acinar to ductal metaplasia and PanIN 1 are not systematically recognized by immune system in contrast to higher grade PanIN. Moreover, the earliest dysplasia still drives immunosuppressive mechanism through LGALS9 expression such that CD8+ T-cell encounter immunosuppression before immunological activation.

Interestingly, we have shown, with the scoring chart we have developed, an increase in the total area of PanIN in untreated mice, and the pathologist’s analysis reveals that untreated mice also have a higher grade of PanIN (Grade II). In such a situation, how can we explain the impact of the anti-galectin-9 treatment on PanIN progression independently of an active immune response?

Galectin-1 and 3 have been broadly studied in PDAC and also in KRAS-driven mouse model, and studies indicate that they are deeply involved in ADM and PanIN formation. Different mechanisms have been described either directly through acini and tumor cells or indirectly through immune cell and pancreatic stellate cells. Indeed, we thought that LGALS9 could be directly implicated in tumor promotion and ADM. This hypothesis is supported by several publications showing that LGALS9 binds to CD44 in the presence of Transforming Growth Factor Beta (TGFβ) to induce clustering of CD44 and the TGFβ receptor and thus amplify TGFβ signalling (39). In pancreatic cancer, the crucial role of CD44/TGFβ receptors (TβRI and TβRII) signalling is well admitted not only in epithelial-to-mesenchymal transition but also in the earlier ADM step. This hypothesis is very likely with regard to the ability of other galectins to bridge different receptors to promote carcinogenesis (70–72). The hypothesis that LGALS9 mediates TGFβ and CD44 signalling in KRAS-driven carcinogenesis will need further investigation.

## Conclusion

Our results confirmed the major importance of galectin-9 in PDAC. Among others, we show that galectin-9 is expressed early during pancreatic carcinogenesis as early as ADM and is correlated with Treg infiltration. Furthermore, galectin-9 neutralization severely impaired both Treg rates during carcinogenesis and PanIN formation, thus making galectin-9 a high-value target. We cannot exclude synergy between the different galectins in Treg-mediated immunosuppression and PanIN formation, as the functions of galectins have been shown to overlap in many contexts, including PDAC. Therefore, tumors can compensate the blockade effects of selective galectin inhibitors by upregulating another galectin with similar functions. However, antagonizing only galectin-9 was sufficient to impede PanIN progression and Treg increase in this context. Given the importance of galectin-9 in anti-PD1 resistance and its role in pancreatic carcinogenesis, tolerogenic reprogramming and adaptive immune suppression targeting galectin-9 and PD-1 may be a promising strategy to unblock the immune response in pancreatic cancer.

## Data availability statement

The datasets presented in this study can be found in online repositories. The names of the repository/repositories and accession number(s) can be found below: https://www.ncbi.nlm.nih.gov/, GSE53659.

## Ethics statement

The animal studies were approved by Comité Ethique Expérimentation Animale Nord Pas-de-Calais, #AF042008 and #00422.02. The studies were conducted in accordance with the local legislation and institutional requirements. Written informed consent was obtained from the owners for the participation of their animals in this study.

## Author contributions

AQ: Conceptualization, Data curation, Formal analysis, Investigation, Writing – original draft, Writing – review & editing. RM: Formal analysis, Writing – original draft, Investigation. BD: Investigation, Methodology, Writing – original draft. AK: Formal analysis, Investigation, Writing – original draft. EW: Investigation, Methodology, Software, Writing – original draft. EL: Methodology, Validation, Writing – original draft. OM: Data curation, Formal analysis, Methodology, Project administration, Validation, Visualization, Writing – original draft, Writing – review & editing. NJ: Methodology, Resources, Validation, Writing – review & editing. IS: Resources, Validation, Writing – review & editing. ND: Conceptualization, Funding acquisition, Project administration, Supervision, Validation, Visualization, Writing – original draft, Writing – review & editing.

## Supplementary material

Supplementary Figure 1 Experimental overview of the study. **(A)** Determination of the significance of both peripheral and pancreatic Treg and Galectin-9 during the progression of preneoplastic lesions of the pancreas (PanIN) in the KRAS^G12D^ model as compared to wild-type mice. **(B)** Ex-vivo assessment of an anti-galectin-9 monoclonal antibody in C57Bl/6 murine models targeting murine regulatory T lymphocytes and galectin-9. **(C)** *In vivo* evaluation of anti-galectin-9 immunotherapy on early pre-neoplastic stages of pancreatic adenocarcinoma in KRAS^G12D^ mice.

Supplementary Figure 2 CD8 T cell infiltration in KC mice treated with isotype vs. anti-LGALS9.ImmunoHistChemical staining of CD8 T cells in either 1gG1K isotype versus anti-LGALS9 treated KC mice **(A)**, and relative image analysis of CD8+ T cell infiltration either 1gG1 isotype (KC-Iso) versus anti-LGALS9 (KC Anti-Gal9) treated KC mice **(B)**.
