# Supplementary figures and images for: A novel anti-galectin-9 immunotherapy limits the early progression of pancreatic neoplastic lesions in transgenic mice
Source: Front Immunol. 2023 Nov 30;14:1267279. doi: 10.3389/fimmu.2023.1267279 (PMC10720041; doi:10.3389/fimmu.2023.1267279)

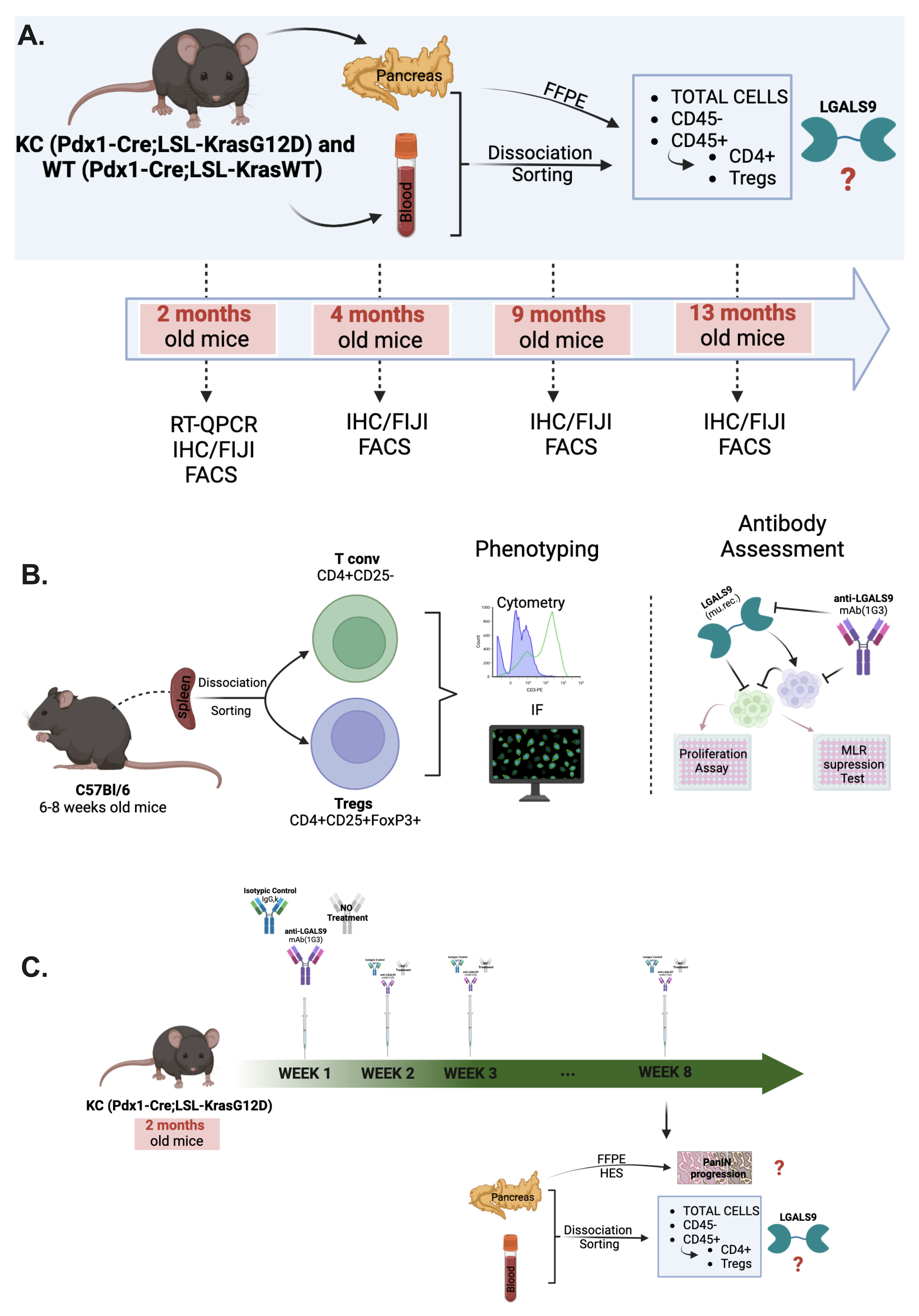

Supplement: Supplementary Figure 1 — Experimental overview of the study. (A) Determination of the significance of both peripheral and pancreatic Treg and Galectin-9 during the progression of preneoplastic lesions of the pancreas (PanIN) in the KRASG12D model as compared to wild-type mice. (B) Ex-vivo assessment of an anti-galectin-9 monoclonal antibody in C57Bl/6 murine models targeting murine regulatory T lymphocytes and galectin-9. (C) In vivo evaluation of anti-galectin-9 immunotherapy on early pre-neoplastic stages of pancreatic adenocarcinoma in KRASG12D mice. [file Image_1.tif]

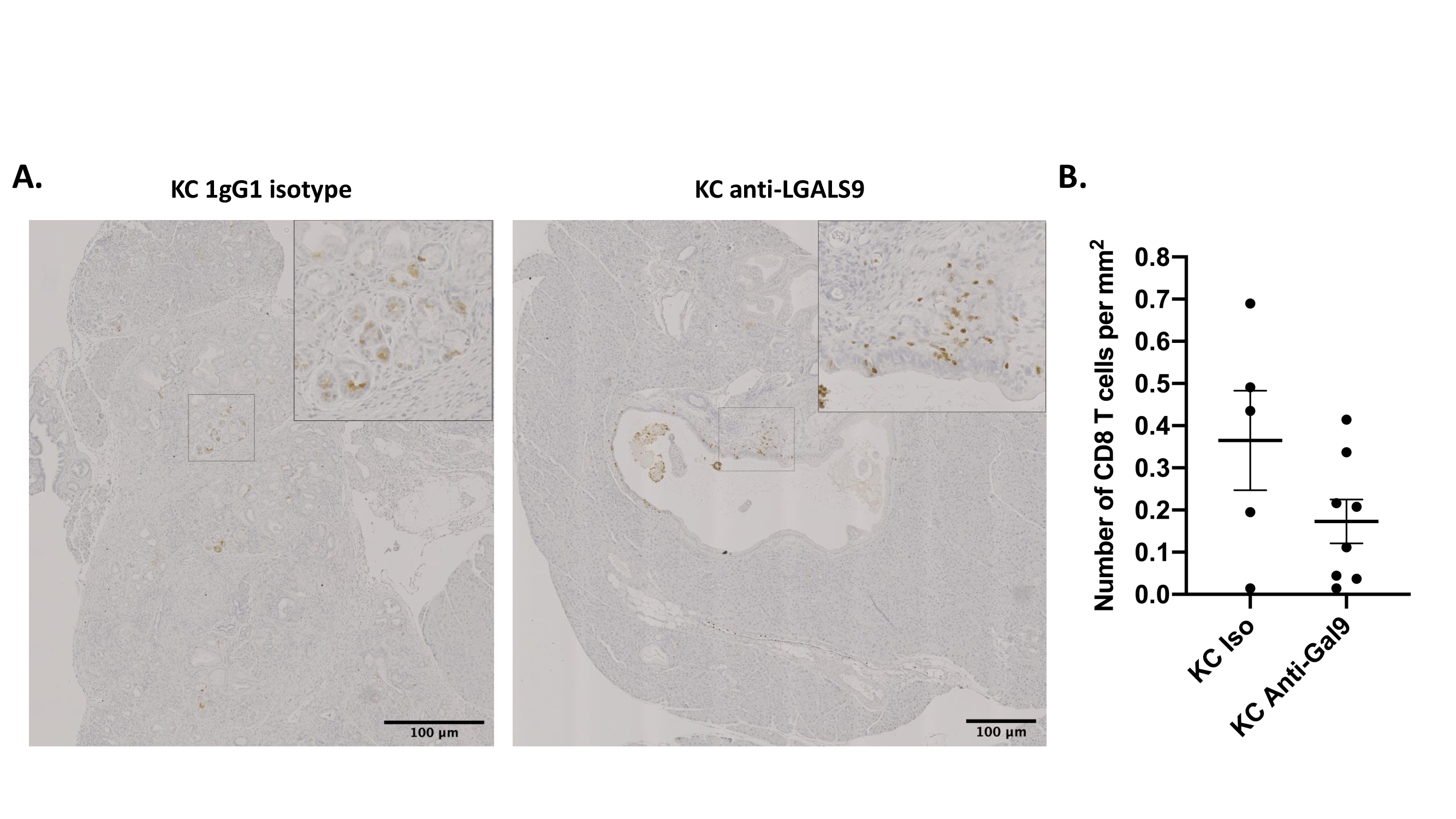

Supplement: Supplementary Figure 2 — CD8 T cell infiltration in KC mice treated with isotype vs. anti-LGALS9.ImmunoHistChemical staining of CD8 T cells in either 1gG1K isotype versus anti-LGALS9 treated KC mice (A), and relative image analysis of CD8+ T cell infiltration either 1gG1 isotype (KC-Iso) versus anti-LGALS9 (KC Anti-Gal9) treated KC mice (B). [file Image_2.tiff]
